# Book Review

**DOI:** 10.1120/jacmp.v3i3.2570

**Published:** 2002-06-01

**Authors:** Peter R. Almond

**Affiliations:** ^1^ Department of Radiation Physics MD. Anderson Cancer Center University of Texas 1515 Holcombe Blvd. Houston TX 77030

## Abstract

**Shielding techniques for radiation oncology facilities** Patton H. McGinley, Medical Physics Publishing, Madison, WI 2002. 184pp. Price: $65.00.

PACS number(s): 87.52.–g

This is the second edition of this book, which was first published in 1998. The first edition was well received and met a critical need in the area of shield calculations and room design for radiation oncology facilities. Up until that time calculations and room design were based on several reports published over 20 years ago by the National Council of Radiation Protection and Measurement (NCRP). Because of advances in radiation sources and techniques, shielding and room design considerations for radiation oncology, not covered by the NCRP reports, have arisen. McGinley's first book filled in the gaps, put all the information in one book instead of the several NCRP reports, and was specifically for radiation oncology. The NCRP reports were more general and information for radiation oncology facilities had to be extracted from them.

This new edition addresses issues that have arisen since the first edition, including gamma knife rooms, CT simulator rooms, and questions raised by intensity modulated radiation therapy (IMRT). Because this book is suitable as a textbook there are homework problems and sample calculations and these have been upgraded, along with some figures, tables, and references from the previous edition. This edition will clearly be as helpful as the first edition.

As a handbook for the professional doing room designs and calculations, this is an invaluable resource. However, I do have some concerns about the use of this volume as a textbook. In Peter Biggs'^1^ excellent review of the first edition of this book, he points out the omission of any discussion of C60o units in the historical edition. The shielding consideration for C60o units is clearly of historical interest and 60CO units are still in use around the world, while the betatrons are all but extinct. Why were betatrons mentioned and not C60o?

Biggs also points out questions concerning quantities and units. This is an area that can be very confusing in shielding calculations, and it is imperative to use consistent quantities and units through the calculations. This book would be enhanced with a section on this subject, explaining what is used in the calculations and the pitfalls that can arise. Uniform SI units are not used throughout, and conversions may be needed. European building plans are scaled in meters, American plans in feet, for example, and it is very easy to make mistakes.

I would also like to have seen a section on the derivation of the basic equations, especially Eq. (2.1):

$$B_{x}=\frac{P{\left(d_{pri}\right)}^{2}}{WUT},$$

which appears without any introduction. To the veteran this equation may seem obvious but to the student it is not. An explanation of how this equation is derived can teach a student a lot about the philosophy behind shielding calculations and room design.

Curiously the author states in the historical section that “The basic calculation techniques for shielding are similar to those given in NBS Handbook 60 except as noted … ” and he refers to Handbook 60 several other times in this section. But NBS Handbook 60 is not shown in the references at the end of the book. Further, it is not referenced in the NCRP Report Nos. 49, 51, and 79, which are the basis for the material in this book.

Strangely, no mention is made of computer programs for shielding calculations. Since they are readily available it would have been helpful to have a section on them, explaining the necessity of spot checking the computer results with hand calculations. The book would also be enhanced with the addition of a table of content for tables and graphs and a section on definitions. Not everyone speaks the same language.
